# Can big data solve a big problem? Reporting the obesity data landscape in line with the Foresight obesity system map

**DOI:** 10.1038/s41366-018-0184-0

**Published:** 2018-09-21

**Authors:** Michelle A. Morris, Emma Wilkins, Kate A. Timmins, Maria Bryant, Mark Birkin, Claire Griffiths

**Affiliations:** 1 Leeds Institute for Data Analytics and School of Medicine, University of Leeds, Leeds, LS2 9JT UK; 2 School of Sport, Leeds Beckett University, Leeds, LS6 3QQ UK; 3 School of Sport and Exercise Science, College of Social Science, University of Lincoln, Lincoln, LN6 7TS UK; 4 Leeds Institute of Clinical Trials Research, University of Leeds, Leeds, LS2 9JT UK; 5 Leeds Institute for Data Analytics and School of Geography, University of Leeds, Leeds, LS2 9JT UK

## Abstract

**Background:**

Obesity research at a population level is multifaceted and complex. This has been characterised in the UK by the Foresight obesity systems map, identifying over 100 variables, across seven domain areas which are thought to influence energy balance, and subsequent obesity. Availability of data to consider the whole obesity system is traditionally lacking. However, in an era of big data, new possibilities are emerging. Understanding what data are available can be the first challenge, followed by an inconsistency in data reporting to enable adequate use in the obesity context. In this study we map data sources against the Foresight obesity system map domains and nodes and develop a framework to report big data for obesity research. Opportunities and challenges associated with this new data approach to whole systems obesity research are discussed.

**Methods:**

Expert opinion from the ESRC Strategic Network for Obesity was harnessed in order to develop a data source reporting framework for obesity research. The framework was then tested on a range of data sources. In order to assess availability of data sources relevant to obesity research, a data mapping exercise against the Foresight obesity systems map domains and nodes was carried out.

**Results:**

A reporting framework was developed to recommend the reporting of key information in line with these headings: Background; Elements; Exemplars; Content; Ownership; Aggregation; Sharing; Temporality (BEE-COAST). The new BEE-COAST framework was successfully applied to eight exemplar data sources from the UK. 80% coverage of the Foresight obesity systems map is possible using a wide range of big data sources. The remaining 20% were primarily biological measurements often captured by more traditional laboratory based research.

**Conclusions:**

Big data offer great potential across many domains of obesity research and need to be leveraged in conjunction with traditional data for societal benefit and health promotion.

## Introduction

The basic drivers of obesity are simple (more energy consumed than expended); however, the aetiology is complex. It is now widely accepted that multiple factors, including physiological, social and environmental, act synergistically to drive obesity. These factors are often described as the ‘obesogenic’ environment (an environment that hinders sufficient physical activity and promotes excessive intake of food, thereby making obesity more likely). This may explain the limited success – at a population level – of traditional approaches to obesity prevention and management, which have tended to focus on behavioural, educational and pharmacological factors. For this reason, many researchers and policymakers are now advocating for ‘whole systems’ approaches to obesity prevention and management, which promote integrated systems to address obesity, rather than focusing on risk factors in isolation [1–3].

In 2007, the Foresight Report—the most comprehensive UK investigation into obesity and its causes [1]—described obesity as a “complex web of societal and biological factors that have, in recent decades, exposed our inherent human vulnerability to weight gain”. The investigation produced an obesity system map, with energy balance at its centre. Around this, over 100 variables are split into seven domains that are thought to directly or indirectly influence energy balance.

Knowing that individual (e.g., genetics, age, gender and ethnicity), social (income, education, area deprivation) and area factors (e.g., access to fast food, street connectivity) contribute towards obesity is useful [4–6] and has identified key areas to target prevention and/or treatment. However, the key is understanding the interplay between these factors, which is currently lacking. The complex, nonlinear and unpredictable relationships of how systems interact will offer insight into the development and evaluation of systems based approaches, moving away from siloed thinking [7]. Data required to fill gaps in traditional resources and to enable research using a whole systems approach are inherently difficult to collect, especially on a large scale. For this reason, new and emerging data sources are increasingly gaining attention.

Internationally, a broader understanding of risk factors for obesity and increasing awareness of the social determinants have led to recognition of the need for more comprehensive, cross sectoral strategies to tackle obesity [8].

The preceding paper in this series [9] reviewed how ‘found’ data sources, often referred to as ‘big data’, have been utilised in the literature to better understand obesity. Data on our activity, behaviour and location, from sources as diverse as smart motorways, social media, store loyalty cards and consumer organisations, have been shown to offer fruitful research opportunities, contributing in ways where traditionally sourced research data perhaps could not.

This second paper from the Economic and Social Research Council (ESRC) Strategic Network for Obesity builds on this description of how ‘big’ or ‘found’ data has been used to date, and considers the future potential of these data to enhance a ‘whole systems’ understanding of obesity. Identifying new types of data becoming available and mapping these onto the domains defined in the Foresight Obesity System Map should reveal the extent to which such data may be capable of addressing the whole system.

One challenge that potentially precludes the use of big data to their full potential is a lack of awareness and understanding around what data exist. Aspects of these new big data sources, such as the volume, variety, velocity and veracity are often challenging to conceptualise and capture. It is therefore also crucial that ways are found to communicate the potential (and limitations) of new, as yet untapped, data sources, across disciplines and sectors, to facilitate the move towards a whole systems approach to obesity.

In this paper, we aimed to:

1. Develop a framework in which to effectively report big data for use under a whole systems obesity lens.
2. Use the new framework to report indicative exemplar data types in line with the Foresight obesity system map domain areas.
3. Identify big data sources for use in whole systems obesity research and map these against the Foresight obesity systems map.
4. Discuss key challenges associated with using new and large data sources to analyse obesity from a whole systems perspective.

## Methods

This paper is one output from a collaborative network of academic researchers, industry partners, charity representatives and members from the public sector. We convened 40 members and hosted 5 network meetings between 2015 and 2017 Editorial is 2018IJO00672R. During these meetings, members shared: experiences of using big data for obesity research, knowledge of suitable data sources, and expert opinion on how to optimise this wealth of data. For the purpose of this paper, big data were defined synonymously with ‘non-traditional’ data; in other words, any data not collected specifically for academic research purposes.

Synthesis of expert opinion on optimising data, culminated in the development of a reporting framework. The purpose of this framework was to outline a structure for reporting the features of big data in obesity research, although its application may be valid outside the obesity research area.

The reporting framework was then applied to eight exemplar data sets, to demonstrate its usefulness in communicating important data details. The foresight obesity systems map was indexed with domain and node identifiers (presented in the supplementary material). We use indicative use cases to present the relevant foresight nodes within the exemplar reporting.

In order to illustrate the potential scope and depth of big data, a list of potential data sets available in the UK, were mapped against the nodes of the Foresight obesity systems map. The objective was not to perform a comprehensive audit, which would quickly become outdated, but rather to demonstrate the potential value and opportunity of big data as a resource in understanding the obesity system. The list of data sets was thus a convenience sample, based on data sets that were familiar to network members. The mapping exercise was supplemented with one more traditional and comprehensive dataset, the UK Biobank cohort, to highlight how combination of different types of data might be used together.

For other application areas (i.e., not obesity) this exercise could be repeated with reference to another domain specific theoretical framework.

## Results

### BEE-COAST framework

ESRC Strategic network for obesity members agreed that, for all new data sources, it is essential to provide some background (**B**) on the history and purpose of how and why the data are generated, including key features of the data. This is especially important when data are used in a context for which they were not initially collected. Detailed description of the Elements (**E**) of the data that are required for others to fully understand their potential application. These Elements encompass detailed Content (**C**), Ownership (**O**), level of aggregation, for example individual, neighbourhood, regional or national (**A**), conditions related to Sharing (**S**) and Temporality (**T**) of the data. Finally, these datasets should be illustrated using Exemplars (**E**) to include the format of the data and indicative use cases (Table 1).

**Table 1:** BEE-COAST framework

**B**ackground	Key features
	History
	Purpose
**E**lements	**C**ontent
	**O**wnership
	**A**ggregation
	**S**haring
	**T**emporality
**E**xemplars	Indicative use cases
	Foresight nodes

### Reporting data sources

Eight exemplar data sources were included in this review as providing valuable data for use in a whole systems obesity research: (i) Ordnance Survey Points of Interest data; (ii) Food Standards Agency food hygiene data; (iii) supermarket loyalty card data; (iv) physical activity applications/wearables; (v) new technologies to record diet; (vi) Acxiom data; (vii) Cameo data from Callcredit and (viii) YouGov data. The features of these datasets are summarised in Table 2 in accordance with the BEE-COAST reporting framework. Collectively, the exemplar data sets were found to map to 56 Foresight nodes, covering all 7 Foresight domains.

**Table 2:** Example data sources reported in the BEE-COAST framework

Ordnance survey (OS) Points of interest (POI) data		
Background	Key features	POI is a dataset detailing over 4 million geographic features (both natural and built) across Great Britain
	History	The dataset is created and maintained by PointX Ltd on behalf of OS, the national mapping agency of Great Britain. PointX is an independent company jointly owned by OS and Landmark Information Group. POI data has been available since 2000, and is updated quarterly (see below)
	Purpose	POI was developed for the purpose of mapping features of public interest in Great Britain. It is has various uses including both administrative (e.g., service provision and emergency planning) and commercial (e.g., driver routing and location based services)
Elements	Content	POI is a dataset detailing over 4 million geographic features (both natural and built) across Great Britain. The scope of features covered is broad, including commercial services, education and healthcare establishments, transportation infrastructure, attractions, and public infrastructure. Of particular relevance to the obesity system, the dataset contains information on food outlets (various classifications), public transportation nodes (e.g., bus stops), formal green spaces (e.g., commons and parks), and sport and recreational facilities. For each feature, the following data are available: - Unique Reference Number - Feature Name - Feature Classification (600 classifications available) - Feature Address - Feature Location (British National Grid coordinates) - Positional Accuracy of Feature Location - Unique Property Reference Number (allows linkage to OS Address Base suite of products) - Topographic ID and version Identifier (allows linkage to OS MasterMap Topography Layer product). - ITN easting, northing, TOID and version identifier (allows linkage to OS MasterMap ITN layer) - Telephone number and/or web address
	Ownership	Ordnance survey
	Aggregation	Data are available at the level of individual features
	Sharing	POI data can be accessed for free online via the EDINA Digimap website using an educational institution login. However, use of the data via this means is restricted to ‘Educational Use’ and/or limited ‘Administrative Use’, as defined by Ordnance Survey’s end user agreement. Data can be shared with others who have entered into the end user agreement/a data handlers’ agreement with Ordnance Survey. Less restrictive access to the data can be purchased at a cost
	Temporality	A new version of POI is released every quarter. EDINA Digimap hold previous versions of POI back to March 2015. With each new release, OS publish details on the changes that have been made as compared to the previous release. Note, feature classification codes have also changed over time (last update at time of writing: January 2013)
Exemplars	Indicative use cases	POI can be used to characterise access to local amenities relating to diet and physical activity such as food outlets [23], and sport and recreational facilities [24]
	Foresight nodes	4.2 Opportunity for team based activity 4.3 Access to opportunities for physical exercise 4.6 Reliance on labour saving devices and services 4.9 Opportunity for un-motorised transport 4.11 Dominance of motorised transport 4.13 Walkability of living environment 7.4 Food exposure, 7.5 Food abundance, 7.7 Convenience of food offerings, 7.8 Food variety

Food standards agency (FSA) food hygiene data		
Background	Key features	FSA data contains locational, functional (i.e., business type) and hygiene rankings information on food businesses in the UK
	History	Under UK law any business intending to conduct ‘food operations’ (including selling, cooking food, storing, handling, preparing or distributing food) must register their business with the environmental health department of their Local Authority (LA). This is then used by the environmental health team to conduct food hygiene inspections and enforce food law. The register is updated by a LA when a business registers its intention to conduct food operations, and businesses are removed when registered businesses inform a LA of their intention to terminate food operations. Data are also updated when environmental health officers conduct food hygiene inspections. The frequency of such inspection will depend on the initial food hygiene rating assigned to the business
	Purpose	As above in history
Elements	Content	Data are available for all LA that are participating in the Food Hygiene Rating Scheme (FHRS) in England, Northern Ireland and Wales, or the Food Hygiene Information Scheme (FHIS) in Scotland. Participating LAs are listed on the Food Standards Agency website. Presently, all LA in the UK participate in the scheme. Datasets are downloadable separately for each LA. Each dataset contains information on: - Business name - Business type (13 classifications, including ‘Pub/Bar/Nightclub’, ‘Restaurant/Café/Canteen’, ‘Retailers–Supermarkets/Hypermarkets’, ‘Retailers–other’ and ‘Takeaway/Sandwich Shop’) - Business address - Food hygiene ratings and last inspection date - Longitude and latitude
	Ownership	Local authorities
	Aggregation	Data are available at the level of individual businesses
	Sharing	Data are freely available online via the Food Standards Agency website as part of the UK Government’s open data initiative. There are no restrictions as to the use of the data
	Temporality	The FSA website pulls data on a daily basis from LA food hygiene ratings databases. There is no information on how regularly the LA themselves update their databases, and this is likely to vary between LA. Correspondence with an environmental health officer from one LA, for example, indicated that their data were updated fortnightly. Data on the FSA website are overwritten with each daily update, and thus no historical data are available
Exemplars	Indicative use cases	Data can be used to characterise access to food outlets [25, 26] and to assess the quality/acceptability of food offerings within an area (via hygiene ratings)
	Foresight nodes	7.4 Food exposure, 7.5 Food abundance, 7.7 Convenience of food offerings, 7.8 Food variety

Supermarket loyalty card data		
Background	Key features	Transactional records for food and drink purchases (and everything else you can buy in a supermarket)
	History	Traditionally these data are collected for the card holder to gain points on their purchases within a given store. Retailers use the data to target promotions and marketing
	Purpose	As above in history
Elements	Content	Example data fields: - Customer ID (or pseudoID) - Customer home address aggregated to an area level - ID for supermarket address where purchase made - Food type purchased: e.g., avocado - Food group purchased: e.g., produce - Number of items purchased in supermarket - Cost of items purchased in supermarket - Number of items purchased online - Cost of items purchased online - Number of items purchased in convenience store - Cost of items purchased in convenience store
	Ownership	Supermarket or the loyalty card provider if different
	Aggregation	Individual data Geographic identifier–Output area
	Sharing	Currently on a project by project basis. Some data available via the Consumer Data Research Centre (CDRC)
	Temporality	Date and time of purchase available
Exemplars	Indicative use cases	Many examples to date relate to store location planning by major supermarkets, for example demand for grocery retailers in tourist areas, determined by store loyalty card transactions [27]
	Foresight nodes	1.8 Media consumption 1.11 Exposure to food advertising 1.16 Smoking cessation 2.10 Use of medicines 5.7 Level of available energy 5.12 Reliance of pharma remedies 5.20 Quality and quantity of breastfeeding and weaning 6.1 Purchasing power 6.4 Demand for health 6.8 Desire to maximise volume 6.9 Desire to differentiate food offerings 6.11 Desire to minimise costs 6.12 Standardisation of food offerings 6.13 Market price of food offerings 6.17 Societal pressure to consume 7.4 Food exposure, 7.5 Food abundance 7.6 De-skilling 7.7 Convenience of food offerings 7.8 Food variety 7.9 Alcohol consumption 7.11 Energy density of food offerings 7.12 Fibre content of food and drink 7.13 Portion size 7.14 Demand for convenience 7.16 Nutritional quality of food and drink 7.1 Force of dietary habits

Physical activity applications/wearables		
Background	Key features	Real-time or near to real time recording physical activity. Often Global Positioning System (GPS) point data from the phone or app in addition to detailed information from the device. This will likely include information on the duration, intensity, time and place of the activity. Some of the more basic step counters may only include indication of total steps
	History	These devices have become increasingly popular in recent years for personal monitoring of physical activity. Opportunity to earn rewards e.g., Bounts or Pru vitality can be motivating. Opportunity for gamification, or for joining up with friends to challenge each other provide further motivation
	Purpose	To monitor personal physical activity levels
Elements	Content	Example: Bounts - Serial number to identify records in the report - UserID (or pseudo id) - Date and time - App source - Distance travelled (m) - Activity type - Activity duration (s) - Number of steps - MYZONE Effort Points–calculated using the MYZONE system which converts heart rate, calories and time exercising into points - Average speed km/h - First four digits of post code - Gender - Year of birth - GPS point data – latitude, longitude, altitude, accuracy, location type, course, speed
	Ownership	The individual. Access at scale is often via the technology company owner
	Aggregation	Data are at the level of an individual. However identifiers are at an aggregated area level. Fine grain GPS estimates
	Sharing	Bounts data available via the CDRC. This includes data from other fitness devices streamed via the Bounts App. Data from other sources available at a monetary cost e.g., Strava
	Temporality	Bounts data has GPS point data for every 20 minutes throughout the day for data collected by the app installed on a phone. These data are downloaded daily to the Consumer Data Research Centre (CDRC)
Exemplars	Indicative use cases	Prior to the use of new types of activity trackers, assessing the reliability of the data generated by these devices is essential. Evaluation of the popular Fitbit tracker for use in health care monitoring is one example of this [28]
	Foresight nodes	3.1 Physical activity 3.2 Functional fitness 3.3 NEAT non-volitional activity 3.4 Level of recreational activity 3.5 Level of domestic activity 3.6 Level of occupational activity 3.7 Level of transport activity 4.2 Opportunities for team based activity 4.3 Access to opportunities for Physical exercise 4.4 Cost of physical exercise 4.10 Ambient temperature 4.12 Dominance of sedentary employment 4.13 Walkability of living environment 7.4 Food exposure

Web-based or smartphone apps to record diet		
Background	Key features	Using new technologies to record diet offer two new key features: opportunity to select from a wide range of food and beverage products and a timely in depth nutrient breakdown of foods recorded as consumed
	History	Traditionally recording of diet has been done through paper based questionnaires and diaries which are burdensome for participants to complete and for researched to code in nutrient composition software. Nutrient composition software typically only include nutrient breakdown for ~3200 foods, whereas tools like myfood24 offer nutrient composition of ~45000 food at the push of a button
	Purpose	New technologies enable timely recording of diet for personal use and for research purposes
Elements	Content	Self-reported dietary consumption including elements such as: meal slot, time of day, branded and/or generic items, scanned unique product codes (UPC; ‘bar codes’), portion size, own recipes, photos of meals, nutrient composition of foods
	Ownership	The individual and the technology company
	Aggregation	Individual level – nutrient summary information or a full breakdown (120 nutrients). myfood24 will provide the individual’s region of residence. Certain phone apps will likely include some GPS point data
	Sharing	Depends on the technology
	Temporality	Multiple entries are likely depending of the type of use by the individual
Exemplars	Indicative use cases	The MyMealMate app has been evaluated for use in weight loss. And is available for download for Android and IOS. Development, usability and relative validity of myfood24 has been well documented [29, 30]. The tool is available for research purposes currently. The public can access the tool via: www.myfood24.org
	Foresight nodes	4.3 Access to opportunities for physical exercise 5.20 Quality and quantity of breastfeeding and weaning 6.1 Purchasing power 6.4 Demand for health 6.8 Desire to maximise volume 6.9 Desire to differentiate food offerings 6.11 Desire to minimise costs 6.12 Standardisation of food offerings 6.13 Market price of food offerings 6.17 Societal pressure to consume 7.1 Force of dietary habits 7.4 Food exposure 7.5 Food abundance 7.6 De-skilling 7.7 Convenience of food offerings 7.8 Food variety 7.9 Alcohol consumption 7.11 Energy density of food offerings 7.12 Fibre content of food and drink 7.13 Portion size 7.14 Demand for convenience 7.16 Nutritional quality of food and drink

Cameo data from Callcredit		
Background	Key features	Geodemographic classification data
	History	Cameo is a suite of products which have been developed by a commercial organisation. A geodemographic classification was first developed from the 1991 Census (originally ‘Neighbours and Prospects’). The suite has been developed to include a range of classifications (e.g., Cameo Income, Green and Ethical). International classifications have been produced in a number of countries
	Purpose	Cameo has been developed as a commercial product for targeted marketing and credit scoring. Government and public service organisations are also regular users of this and similar competing technologies
Elements	Content	Data are synthesised from a variety of sources, including census data, shareholder registers, house prices, expenditure surveys and corporate data. The product suite covers (many) major domains ranging from holiday preferences and shopping habits to leisure activities and technology awareness. Indicators with direct relevance to obesity include health club membership, participation in active sports and physical exercise, attitudes to health (‘slimmers’, ‘health conscious’), and propensity to visit pubs and restaurants
	Ownership	Cameo data are the property of Callcredit, a commercial organisation based in Leeds, UK with a US parent
	Aggregation	Profiles are commonly available for Lower Super Output Areas (LSOAs) as well as higher geographies such as postal sectors, local authorities and regions. Data may be provided for individual postcodes or even household profiles subject to confidentiality, anonymization and relevant ethical and legal considerations
	Sharing	CDRC has a licence to access core products from the Cameo suite. Applications for use from individual researchers and groups is subject to a Research Approvals Process (data.cdrc.ac.uk). Specific data fields are potentially available subject to the presentation of an appropriate ‘business case’
	Temporality	Some Cameo profiles are anchored in 2011 Census data but are continually updated using longitudinal data about customers, shareholders, voters and so on. Most datasets are updated annually
Exemplars	Indicative use cases	Cameo has been used in characterisation of obesity for neighbourhoods in the UK, US and Australia [31]. Neighbourhood classification has been used as a device for health care resource allocation for many years [32, 33], and in a variety of other applications
	Foresight nodes	1.1 Education 1.2 Acculturation 1.3 Media availability 1.4 Availability of passive entertainment options 1.8 Media consumption 1.11 Exposure to food advertising 1.12 TV watching 6.1 Purchasing power 6.10 Female employment 6.15 Level of employment 6.16 Pressure for growth and profitability 6.17 Societal pressure to consume

YouGov		
Background	Key features	Self-reported data from opinion polls
	History	YouGov provides self-reported data from opinion polls which are collected four times each year from a large panel of 250,000 adults. The questions in the survey are a combination of fixed topics and commissioned content. The themes are extremely wide ranging. A complete catalogue of available data resources may be obtained on request from the data owner
	Purpose	Data were originally collected as a basis for political polls (under the organisation’s original name of Gallup). Commercial and social questions have been developed more recently
Elements	Content	Data spans many thematic areas including consumers, digital, politics, public services, brand profiles, financial services and sports
	Ownership	Data are generated and maintained by YouGov on a commercial basis
	Aggregation	Data are available as cross-classified individual responses which are coded down to a geography of 400 + local authority areas. Demographics are coded by broad categories e.g., gender, age (five groups), social class (six groups)
	Sharing	The CDRC has a licence for data in three key areas of mobility, retail and sustainability. The variables relevant to health include product consumption (e.g., meat, vegetables, alcohol, carbonated drinks, confectionery and snacks); eating habits (self-classified) and concerns about food (e.g., salt, sugar, fats, gluten). Commissioned tables can potentially be generated at a modest but commercial rate
	Temporality	Data are updated quarterly.
Exemplars	Indicative use cases	YouGov data have been regularly used [34]. Current work is considering the relationship between supermarket accessibility and electronic delivery of groceries, in which individual level choices are a useful feature.
	Foresight nodes	1.1 Education 1.5 Sociocultural valuation of food 2.9 Demand for indulgence/compensation 3.7 Level of transport activity 4.6 Reliance on labour saving devices and services 6.2 Pressure to improve access to food offerings 6.3 Pressure to cater for acquired tastes 6.4 Demand for health 7.1 Force of dietary habits 7.3 Tendency to graze 7.4 Food exposure 7.5 Food abundance 7.6 De-skilling 7.7 Convenience of food offerings 7.8 Food variety 7.9 Alcohol consumption 7.11 Energy density of food offerings 7.12 Fibre content of food and drink 7.13 Portion size 7.14 Demand for convenience 7.16 Nutritional quality of food and drink

Acxiom		
Background	Key features	Self-reported data from voluntary consumption surveys
	History	Acxiom is a very large poll collected in the order of one million returns every year. The data are primarily sourced from product guarantees and media (e.g., newspaper) inserts
	Purpose	Data are from market research and widely used in marketing, advertising and also within local government
Elements	Content	Data includes basic demographics (age, gender, household composition) but also income and expenditure attributes. Relevant to obesity, it includes consumption profiles and lifestyle attitudes including sports and leisure pursuits. The content of irregular commissioned tables ranges from interest in holidays in Yorkshire to purchase of pet foods
	Ownership	Acxiom is a private company which is now part of the VNU multi-media transnational corporation. The majority of the data owned by Acxiom are only accessible through commercial licence
	Aggregation	Data are at individual level, coded to unit postcodes and classified by demographics and other self-reported categories for activity, behaviour and consumption variables
	Sharing	Income and household composition profiles for unit postcodes (1.2 million streets) are licensed for the use of CDRC and its partners. Data relate to calendar year 2014
	Temporality	Data have been collected since at least 2005, with many variables captured on a recurrent basis. Composition of the sample varies from year to year according to responsiveness of consumers and their exposure to the questionnaires
Exemplars	Indicative use cases	Exploration of the Acxiom data in the context of household migration has been undertaken by Thomas (2014) [35]. Use of the data in the context of retail consumption in times of austerity and the “credit crunch” have been considered by Thompson (2013) [36] and Clarke (2015) [37]. These academic studies have explored and reweighted for skews and variable quality of the individual returns
	Foresight Nodes	1.1 Education, 1.3 Media availability, 1.4 Availability of passive entertainment options, 1.8 Media consumption, 1.11 Exposure to food advertising, 1.12 TV watching, 1.16 Smoking cessation, 2.2 Face to face social interaction, 3.1 Physical activity, 3.4 Level of recreational activity, 3.5 Level of domestic activity, 3.6 Level of occupational activity, 3.7 Level of transport activity, 4.6 Reliance on labour saving devices and services, 4.11 Dominance of motorised transport, 6.1 Purchasing power, 6.10 Female employment, 6.15 Level of employment

### Mapping data sources to Foresight domains and nodes

The list of data sources and how these map onto the Foresight nodes can be seen in Table 3. Overall, 86/108 of the Foresight nodes are covered by at least one big dataset. When traditional cohort data is also included (UK Biobank), this increases to 89/108 nodes.

**Table 3:** Mapping data sources to the Foresight obesity system map

Traditional data	
Cohort study	Foresight nodes
UK Biobank http://www.ukbiobank.ac.uk/data-showcase/	1.1 Education 1.4 Availability of passive entertainment options 1.15 Social rejection of smoking 1.16 Smoking cessation 2.1 Self-esteem 2.2 Face to face social interaction 2.4 Stress 2.10 Use of medicines 3.1 Physical activity 3.2 Functional fitness 3.4 Level of recreational activity 3.5 Level of domestic activity 3.6 Level of occupational activity 3.7 Level of transport activity 4.10 Ambient temperature 4.11 Dominance of motorised transport 5.2 Resting metabolic rate 5.4 Genetic and or epigenetic predisposition to obesity 5.6 Appropriateness of embryonic and foetal growth 5.12 Reliance on pharma remedies 5.13 Reliance on surgical interventions 5.24 Level of fat free mass 6.1 Purchasing power 6.15 Level of employment 7.3 Tendency to graze7.8 Food variety 7.9 Alcohol consumption 7.11 Energy density of food offerings 7.12 Fibre content of food and drink 7.13 Portion size 7.16 Nutritional quality of food and drink

Big Data	
Data type/survey	Foresight nodes
Access-card data (e.g., for entry into gyms, lifts, tube, self-service cycle hire)	3.1 Physical activity 3.4 Level of recreational activity 3.7 Level of transport activity 4.4 Cost of physical exercise 4.2 Opportunity for team based activity 4.3 Access to opportunities for physical exercise 4.6 Reliance on labour saving devices and services 4.9 Opportunity for un-motorised transport 4.13 Walkability of living environment
Census	1.1 Education 3.6 Level of occupational activity 3.7 Level of transport activity 4.11 Dominance of motorised transport 4.12 Dominance of sedentary employment 6.10 Female employment 6.15 Level of employment
Commercial survey data	1.1 Education 1.3 Media availability, 1.4 Availability of passive entertainment options 1.5 Sociocultural valuation of food 1.8 Media consumption 1.11 Exposure to food advertising, 1.12 TV watching 1.16 Smoking cessation 2.2 Face to face social interaction 2.9 Demand for indulgence/compensation 3.1 Physical activity 3.4 Level of recreational activity 3.5 Level of domestic activity 3.6 Level of occupational activity 3.7 Level of transport activity 4.6 Reliance on labour saving devices and services 4.11 Dominance of motorised transport 6.1 Purchasing power 6.2 Pressure to improve access to food offerings 6.3 Pressure to cater for acquired tastes 6.4 Demand for health 6.10 Female employment 6.15 Level of employment 6.16 Pressure for growth and profitability 6.17 Societal pressure to consume 7.1 Force of dietary habits 7.3 Tendency to graze 7.4 Food exposure 7.5 Food abundance 7.6 De-skilling 7.7 Convenience of food offerings 7.8 Food variety 7.9 Alcohol consumption 7.11 Energy density of food offerings 7.12 Fibre content of food and drink 7.13 Portion size 7.14 Demand for convenience 7.16 Nutritional quality of food and drink
Commercial weight-loss services	2.2 Face to face social interaction 5.1 Degree of primary appetite control 5.18 Appropriateness of nutrient partitioning 5.24 Level of Fat Free 7.6 De-skilling 7.9 Alcohol consumption 7.13 Portion size 7.15 Rate of eating 7.16 Nutritional quality of food and drink
Crime survey for England	4.1 Perceived danger in the environment
GP/NHS data	1.16 Smoking cessation 2.4 Stress 2.10 Use of medicines 5.5 Appropriateness of maternal body composition 5.6 Appropriateness of embryonic and foetal growth 5.12 Reliance on pharma remedies 5.13 Reliance on surgical interventions 5.14 Level of infections 5.16 Side effects of drug use 5.19 Appropriateness of child growth 5.20 Quality and quantity of breastfeeding and weaning 7.9 Alcohol consumption
Health/physical activity/diet tracking apps	1.16 Smoking cessation 2.4 Stress 3.1 Physical activity 3.2 Functional fitness 3.3 NEAT non-volitional activity 3.4 Level of recreational activity 3.5 Level of domestic activity 3.6 Level of occupational activity 3.7 Level of transport activity 4.2 Opportunities for team based activity 4.3 Access to opportunities for Physical exercise 4.4 Cost of physical exercise 4.10 Ambient temperature 7.4 Food exposure 4.13 Walkability of living environment 4.12 Dominance of sedentary employment 5.20 Quality and quantity of breastfeeding and weaning 6.1 Purchasing power 6.4 Demand for health 6.8 Desire to maximise volume 6.9 Desire to differentiate food offerings 6.11 Desire to minimise costs 6.12 Standardisation of food offerings 6.13 Market price of food offerings, 6.17 Societal pressure to consume 7.4 Food exposure 7.5 Food abundance 7.7 Convenience of food offerings 7.8 Food variety 7.9 Alcohol consumption 7.11 Energy density of food offerings 7.12 Fibre content of food and drink, 7.13 Portion size 7.14 Demand for convenience, 7.16 Nutritional quality of food and drink 7.6 De-skilling 7.1 Force of dietary habits
Health survey for England	1.1 Education 1.16 Smoking cessation 2.1 Self-esteem 2.3 Individualism 2.4 Stress 2.6 Psychological ambivalence 2.10 Use of medicines 4.12 Dominance of sedentary employment 5.12 Reliance on pharma remedies 5.14 Level of infections 6.6 Pressure on job performance 6.10 Female employment 6.15 Level of employment NB. Specific data collected varies yearly. Foresight mappings are based on 2014 data.
Labour force survey	3.5 Level of domestic activity 4.7 Social depreciation of labour 6.10 Female employment
Mapping data (e.g., from Ordnance Survey)	4.2 Opportunity for team based activity 4.3 Access to opportunities for physical exercise 4.9 Opportunity for un-motorised transport 4.11 Dominance of motorised transport 4.13 Walkability of living environment 7.4 Food exposure 7.5 Food abundance 7.7 Convenience of food offerings 7.8 Food variety
Meteorological data	4.10 Ambient temperature
Social media	1.5 Sociocultural valuation of food 1.6 Importance of the ideal body size image 1.7 Social acceptability of fatness 1.15 Social rejection of smoking 2.2 Face to face social interaction (via ‘check-ins’)
Smart Motorway	3.7 Level of transport activity 4.11 Dominance of motorised transport
Retail sales data	1.8 Media consumption 1.11 Exposure to food advertising, 1.16 Smoking cessation 2.10 Use of medicines 4.6 Reliance on labour saving devices and services 5.7 Level of available energy 5.12 Reliance on pharma remedies 5.20 Quality and quantity of breastfeeding and weaning 6.1 Purchasing power 6.4 Demand for health 6.8 Desire to maximise volume 6.9 Desire to differentiate food offerings 6.11 Desire to minimise costs 6.12 Standardisation of food offerings 6.13 Market price of food offerings 6.17 Societal pressure to consume 7.1 Force of dietary habits 7.5 Food abundance 7.6 De-skilling 7.7 Convenience of food offerings 7.8 Food variety 7.9 Alcohol consumption 7.11 Energy density of food offerings 7.12 Fibre content of food and drink 7.13 Portion size 7.14 Demand for convenience, 7.16 Nutritional quality of food and drink
Sport England	2.2 Face to face social interaction 3.1 Physical activity 3.4 Level of recreational activity 4.2 Opportunity for team based activity 4.3 Access to opportunities for physical exercise 4.4 Cost of physical exercise 4.13 Walkability of living environment
Supermarket loyalty card	1.8 Media consumption 1.11 Exposure to food advertising 1.16 Smoking cessation 2.10 Use of medicines 5.7 Level of available energy 5.12 Reliance of pharma remedies 5.20 Quality and quantity of breastfeeding and weaning 6.1 Purchasing power 6.4 Demand for health 6.8 Desire to maximise volume 6.9 Desire to differentiate food offerings 6.11 Desire to minimise costs 6.12 Standardisation of food offerings 6.13 Market price of food offerings 6.17 Societal pressure to consume 7.1 Force of dietary habits 7.4 Food exposure 7.5 Food abundance 7.6 De-skilling 7.7 Convenience of food offerings 7.8 Food variety 7.9 Alcohol consumption 7.11 Energy density of food offerings 7.12 Fibre content of food and drink 7.13 Portion size 7.14 Demand for convenience 7.16 Nutritional quality of food and drink
Transport surveys	3.7 Level of transport activity 4.11 Dominance of motorised transport 4.13 Walkability of living environment
TV licensing data	1.4 Availability of passive entertainment options 1.12 TV watching

### Absence of mapping to Foresight domains and nodes

Table 4 highlights the areas in which big data are, to the best of our knowledge, not readily available to map against Foresight domains and nodes. We believe that information relating to many of these nodes would typically be generated by research studies, which often recruit, relatively speaking, small number of participants. Whilst participant numbers may be small, the number of data points may be large. Some of the nodes would likely require qualitative research to capture relevant data.

**Table 4:** Unmapped Foresight obesity system map domains and nodes

Foresight domains	Foresight nodes
1. Societal influences	1.9 Peer pressure
	1.10 Conceptualisation of obesity as a disease
	1.13 Perceived lack of time
	1.14 Parental control
2. Individual psychology	2.5 Food literacy
	2.7 Conscious control of accumulation
	2.8 Desire to resolve tension
	2.11 Perceived inconsistency of science based messages
3. Individual physical activity	3.8 Degree of physical education
	3.9 Degree of innate activity in childhood
	3.10 Parental modelling of activity
	3.11 Learned activity patterns in early childhood
4. Physical activity environment	4.5 Sociocultural valuation of activity
	4.8 Safety of un-motorised transport
5. Physiology	5.3 Level of thermogenesis
	5.8 Importance of physical need
	5.9 Effort to acquire energy
	5.10 Tendency to preserve energy
	5.11 Strength of lock-in to accumulate energy
	5.15 Predisposition to activity
	5.17 Level of adipocyte metabolism
	5.18 Appropriateness of nutrient partitioning
	5.21 Level of satiety
	5.22 Degree of optimal gastrointestinal signalling
	5.23 Extent of digestion and absorption
6. Food production	6.5 Effort to increase efficiency of consumption
	6.7 Effort to increase efficiency of production
	6.14 Cost of ingredients
7. Food consumption	7.2 Children’s control of diet
	7.10 Palatability of food offerings

## Discussion

The aim of this paper was to explore the potential role of so-called ‘big data’ in a whole systems approach to obesity. By mapping a small but varied selection of emerging data types onto the Foresight obesity system map, it is apparent that big data span 80% of nodes, and therefore could prove important in providing the breadth and depth of physiological, social, and environmental information needed to simultaneously examine inter-related risk factors for obesity in different populations and across multiple levels. Through this mapping exercise we highlight the wide variety of data which could be better exploited alongside existing research or for new, interdisciplinary, obesity research questions.

Data which span the whole of the obesity system are difficult and time-consuming to collect, particularly on a large scale. Big data have been heralded as a potential solution to this problem, with such data being generated—largely passively—at an ever-increasing rate and across a range of contexts. This is the first time the potential for big data has been evaluated in a whole systems context. Our data audit has shown the potential value of big data within this field. Furthermore, value does not only arise from the advances in research, but financially through reduction in cost of primary data collection, which can result in both researcher and participant burden.

Whilst the broad coverage of big data across the obesity system map offers exciting possibilities for research, it is important to acknowledge that big data are not the complete solution to a whole systems approach. The remaining 20% of nodes on the Foresight obesity system map were not directly featured in our data mapping exercise – for example, genetic and physiological variables relating to appetite control, metabolic rate and predisposition to obesity. Many of these unmapped nodes represent data that are commonly collected in traditional research, and recent large-scale initiatives (e.g., UK Biobank [10, 15] and other ongoing longitudinal cohort studies [11–13]) will continue to contribute important large-scale data. This suggests that big data should be used to supplement and enhance traditional datasets. Indeed, this paper does not advocate for the use of big data in place of traditional data, but rather to complement traditional data and of course be considered in the context of the research [14].

An important aim of this paper was to develop and demonstrate a framework (BEE-COAST) for reporting big data that describes emerging data through a whole systems obesity lens. The framework was shown to successfully summarise and communicate the important features of a number of data sources, including vital information about ownership and sharing, as well as content. It is suggested that this framework should be used to report big data sources used in research. It is also proposed that this framework could be used to develop a reference list of big data sources as a resource for future research, akin to published data resources profiles. Its application may also be valid outside the obesity research area.

While the BEE-COAST framework goes some way to elucidating the available ‘big data’ for obesity research, further data sources still need to be made available to increase coverage of the Foresight obesity system map. Increased multidisciplinary may facilitate this. For example, while our data audit did not highlight much data relating to food production, data are certainly being captured across this domain, for example by satellites, the instrumentation of farms, and data driven control within the manufacturing process. Such sources are not necessarily evident or accessible to a research community around obesity. Interdisciplinary networks such as the ESRC’s Strategic Network for Obesity, and repositories such as the CDRC, provide a long-term opportunity to ameliorate this difficulty.

We have seen that variables as diverse as physical activity behaviours, built environment features, food consumption and choice are all richly captured by emerging sources of data. However, in spite of the existence of these data, we may not yet be in a position to utilise them to their full potential due to restrictions around data access and linkage. While individual-level data exists (e.g., relating to physical activity behaviours), at times these data cannot be released at an individualised level due to confidentiality and anonymity restrictions. Individual-level linkage is only possible with explicit consent. This has implications for data linkage, as data are often released (and thus must be linked) at a neighbourhood level, or larger, rather than at the individual level. Such area-level linkage is less than ideal, as potentially important within-neighbourhood variability is lost, and analyses are subject to bias (e.g., the ecological fallacy). Innovative approaches to data sharing and linkage are needed. As an example, UK Biobank [15] overcomes this problem by releasing individual-level addresses for linkage with other datasets prior to the release of the main cohort data. In this model, the researcher can link the addresses with any secondary datasets, and then return this linked data to UK Biobank to be joined with the cohort data. Finally, the cohort data and linked secondary data are released back to the researcher, with the participant addresses removed. This process was possible because participants explicitly consented to take part in the UK Biobank study, which includes health data linkage. It is important to note that we have not explored the finer details of how such data sources might be linked and harmonised for research purposes.

Where data linkage is often feasible and legal, whether it is ethical to perform such linkages is a wider consideration [16, 17]. For example; users of fitness tracking devices may have consented within their terms and conditions to sharing of their data with trusted partners. However, could they reasonably have expected that these partners would combine these physical activity records with food purchase transactions and their health outcomes? In many cases the benefit to society from such research may be argued to outweigh the risk of identification of the individual, but does that mean we should link data in this way, and would it, or should it, be permitted by research ethics committees responsible for granting ethical approval for research? The role of the ethics committee is essential to protecting the interests of the public and the research community. Should ethical standards not be maintained there is a risk of public outcry, which could prohibit future research of this type. Worse still would be for individual-level information to leak outside the research communities, for example to insurance companies, who might penalise their customers.

In relation to data access, the ownership of data is another key issue. Supermarkets may be keen to share data with academic researchers if there is hope of serious insights into store planning or optimisation of marketing spend. Whether they are also keen to share data to understand negative health consequences from retail sales is a somewhat different proposition. The ability to document the ownership of data sources is not always straightforward. For example; Food Standards Agency (FSA) data may be hosted and accessed via a local authority, but whether local authorities or the FSA are the data owner is debatable. This is likely the case for other data sources accessed via a third party.

These issues around access, linkage and ethics are echoed in the literature: most of the studies published to date that have attempted to utilise ‘found’ data describe challenges relating to these concerns [9]. It is encouraging however, that solutions to these challenges have been found, illustrated by the publication of such studies. Sharing best practice between research teams and organisations, relating to these challenges, presents opportunity to progress with a new type of research more pragmatically and efficiently.

In this paper we have considered how the Foresight obesity system map might be more fully populated through extraction of big data sources. However, the transformative effects of big data are potentially much more wide-ranging. A primary example of this is in the field of Randomised Controlled Trials (RCTs), where there is a growing feeling that a combination of new datasets at scale, perhaps ranging from patient data, hospital outcomes and prescriptions to lifestyle, activity, eating and food purchases might be combined to create a massive population base for future trials. Such an approach could be cost efficient in targeting participants, it would allow substantial samples to be identified for even the rarest conditions, and potentially admit variations between focused sub-groups e.g., within a specific age range or ethnic category. Again, the ability of trials to utilise such data is largely dependent upon the availability and accessibility of individual level data.

Innovative approaches to research questions are required in changing political landscapes and big data presents valuable possibilities. While RCTs are heralded as the gold standard in study design they are not applicable to all research questions. Many research questions relating to obesogenic factors (e.g., social and built environmental variables) are better suited to observational rather than experimental. This challenge is compounded in that big data is generally inherently observational rather than experimental. Thus we may need to look to alternative study designs combined with alternative and innovative methods of analysis. In particular, big data presents valuable possibilities for natural experiments to compare the experience of similar groups under different environmental conditions or subject to different interventions e.g., in different regions [18–21]. Spatiotemporal patterns can be investigated at scale for the first time without the challenges associated with longitudinal cohort study design and follow-up.

The above notwithstanding, a plethora of approaches, methods, metrics and variables are already being used in studies that make cross-comparison difficult - even impossible - and so the search for definitive evidence difficult. It is also important to maintain scientific rigour and a critical perspective and humility; employing *a priori* hypotheses where relevant, or acknowledging hypothesis generation where this alternative is used. Current practices of reporting statistical significance are in urgent need of refreshment because large sample sizes will always produce highly significant results and thus reporting of effect sizes and clinical meaningfulness is essential. Heterogeneity in data collection methods and resulting biases must be considered and acknowledged. This may be further inflated through combining multiple data sources. The use of big data does not preclude the need for validation of findings, whether that is through use data generated from a ‘gold standard’ or using better understood traditional data sources. Multiple big data sources, combined with traditional datasets offer opportunities for cross-validation, which is especially important when findings result from hypothesis generation. Given the many strengths of big data, we may need to accept these limitations as a necessary compromise. However, newly developing machine learning methods, and new strategies for causal inference with observational data may be part of a solution to these challenges [22].

In conclusion, big data offer great potential across many domains of obesity research and need to be leveraged for societal benefit and health promotion. While obesity research and policy have evolved towards a ‘whole systems’ paradigm since the publication of the Foresight Report, they still tend to focus only on small parts of the obesity system in isolation, and fail to consider the interrelationships between different factors. Use of big data could facilitate understanding of the wider determinants of obesity and their interrelations across multiple levels. In turn, this would permit evidence-informed allocation of funds and ultimately optimise return on investment during a period of financial constraint. This is particularly timely in light of the Government’s childhood obesity policy published this year, which, in spite of identifying 14 specific levers for change, found a best-case summary of existing evidence-base to be ‘equivocal’.

## Electronic supplementary material

Foresight domains and nodes
