# Supplementary material for: Can big data solve a big problem? Reporting the obesity data landscape in line with the Foresight obesity system map
Source: Int J Obes (Lond). 2018 Sep 21;42(12):1963–76. doi: 10.1038/s41366-018-0184-0 (PMC6291418; doi:10.1038/s41366-018-0184-0)
Supplement: Supplementary file 1 — Foresight domains and nodes [file 41366_2018_184_MOESM1_ESM.docx]

| **Foresight Domains** | **Foresight Nodes** |
| --- | --- |
| 1. Societal influences | 1.1 Education  1.2 Acculturation  1.3 Media availability  1.4 Availability of passive entertainment options  1.5 Sociocultural valuation of food  1.6 Importance of the ideal body size image  1.7 Social acceptability of fatness  1.8 Media consumption  1.9 Peer pressure  1.10 Conceptualisation of obesity as a disease  1.11 Exposure to food advertising  1.12 TV watching  1.13 Perceived lack of time  1.14 Parental control  1.15 Social rejection of smoking  1.16 Smoking cessation |
| 2. Individual psychology | 2.1 Self-esteem  2.2 Face to face social interaction  2.3 Individualism  2.4 Stress  2.5 Food literacy  2.6 Psychological ambivalence  2.7 Conscious control of accumulation  2.8 Desire to resolve tension  2.9 Demand for indulgence/compensation  2.10 Use of medicines  2.11 Perceived inconsistency of science based messages |
| 3. Individual physical activity | 3.1 Physical activity  3.2 Functional fitness  3.3 NEAT non-volitional activity  3.4 Level of recreational activity  3.5 Level of domestic activity  3.6 Level of occupational activity  3.7 Level of transport activity  3.8 Degree of physical education  3.9 Degree of innate activity in childhood  3.10 Parental modelling of activity  3.11 Learned activity patterns in early childhood |
| 4. Physical Activity Environment | 4.1 Perceived danger in the environment  4.2 Opportunity for team based activity  4.3 Access to opportunities for physical exercise  4.4 Cost of physical exercise  4.5 Sociocultural valuation of activity  4.6 Reliance on labour saving devices and services  4.7 Social depreciation of labour  4.8 Safety of un-motorised transport  4.9 Opportunity for un-motorised transport  4.10 Ambient temperature  4.11 Dominance of motorised transport  4.12 Dominance of sedentary employment  4.13 Walkability of living environment |
| 5. Physiology | 5.1 Degree of primary appetite control  5.2 Resting metabolic rate  5.3 Level of thermogenesis  5.4 Genetic and or epigenetic predisposition to obesity  5.5 Appropriateness of maternal body composition  5.6 Appropriateness of embryonic and fetal growth  5.7 Level of available energy  5.8 Importance of physical need  5.9 Effort to acquire energy  5.10 Tendency to preserve energy  5.11 Strength of lock-in to accumulate energy  5.12 Reliance on pharma remedies  5.13 Reliance on surgical interventions  5.14 Level of infections  5.15 Predisposition to activity  5.16 Side effects of drug use  5.17 Level of adipocyte metabolism  5.18 Appropriateness of nutrient partitioning  5.19 Appropriateness of child growth  5.20 Quality and quantity of breastfeeding and weaning  5.21 Level of satiety  5.22 Degree of optimal gastrointestinal signalling  5.23 Extent of digestion and absorption  5.24 Level of fat free mass |
| 6. Food production | 6.1 Purchasing power  6.2 Pressure to improve access to food offerings  6.3 Pressure to cater for acquired tastes  6.4 Demand for health  6.5 Effort to increase efficiency of consumption  6.6 Pressure on job performance  6.7 Effort to increase efficiency of production  6.8 Desire to maximise volume  6.9 Desire to differentiate food offerings  6.10 Female employment  6.11 Desire to minimise cost  6.12 Standardisation of food offerings  6.13 Market price of food offerings  6.14 Cost of ingredients  6.15 Level of employment  6.16 Pressure for growth and profitability  6.17 Societal pressure to consume |
| 7. Food consumption | 7.1 Force of dietary habits  7.2 Children’s control of diet  7.3 Tendency to graze  7.4 Food exposure  7.5 Food abundance  7.6 De-skilling  7.7 Convenience of food offerings  7.8 Food variety  7.9 Alcohol consumption  7.10 Palatability of food offerings  7.11 Energy density of food offerings  7.12 Fibre content of food and drink  7.13 Portion size  7.14 Demand for convenience  7.15 Rate of eating  7.16 Nutritional quality of food and drink |

**Broad domains and specific nodes of the Foresight Obesity Systems Map (Foresight, 2007).**

FORESIGHT. 2007. Tackling Obesities: Future Choices – Project report.
